# Retraction Note: Immune landscape of periodontitis unveils alterations of infiltrating immunocytes and molecular networks-aggregating into an interactive web-tool for periodontitis related immune analysis and visualization

**DOI:** 10.1186/s12967-024-05812-5

**Published:** 2024-11-04

**Authors:** Xiaoqi Zhang, Qingxuan Wang, Xinyu Yan, Yue Shan, Lu Xing, Minqi Li, Hu Long, Wenli Lai

**Affiliations:** 1 Department of Orthodontics, State Key Laboratory of Oral Diseases, National Clinical Research Center of Oral Diseases, West China Hospital of Stomatology, Sichuan University, 14, Sect 3, Renmin South Rd, Chengdu, 610041 Sichuan China; 2 Department of Bone Metabolism, Shandong Key Laboratory of Oral Tissue Regeneration, School/Hospital of Stomatology, Shandong University, Jinan, Shandong, China

**Retraction Note: Journal of Translational Medicine (2020) 18:438**

10.1186/s12967-020-02616-1

The authors have retracted this article after concerns were raised about the data reported. The dataset used in this analysis, GSE16134, was generated from an archive that contains multiple samples per patient. The analytical approach employed by the authors in this paper did not account for these limitations, and so the authors no longer have confidence in the reliability of their results and conclusions. The authors have been invited to prepare a revised submission containing additional analysis to support their results, which will be submitted for normal peer review.

All authors agree with this retraction.

